# A glance at the application of CRISPR/Cas9 gene-editing technology in cardiovascular diseases

**DOI:** 10.34172/jcvtr.2022.14

**Published:** 2022-06-14

**Authors:** Neda Roshanravan, Helda Tutunchi, Farzad Najafipour, Mohammadreza Dastouri, Samad Ghaffari, Alireza Jebeli

**Affiliations:** ^1^Cardiovascular Research Center, Tabriz University of Medical Sciences, Tabriz, Iran; ^2^Endocrine Research Center, Tabriz University of Medical Sciences, Tabriz, Iran; ^3^Ankara University Biotechnology Institute and SISBIYOTEK Advanced Research Unit, Gumusdere Yerleskesi, Kecioren, Ankara, Turkey

**Keywords:** Cardiovascular Diseases, CRISPR/Cas9, Genome Editing Strategy

## Abstract

Cardiovascular diseases (CVDs) remain major causes of global mortality in the world. Genetic approaches have succeeded in discovery of the molecular basis of an increasing number of cardiac diseases. Genome editing strategies are one of the most effective methods for assisting therapeutic approaches. Potential therapeutic methods of correcting disease-causing mutations or of knocking out specific genes as approaches for the prevention of CVDs have gained substantial attention using genome editing techniques. Recently, the clustered regularly interspaced short palindromic repeats/CRISPR-associated protein 9 (CRISPR/Cas9) system has become the most widely used genome-editing technology in molecular biology due to its benefits such as simple design, high efficiency, good repeatability, short-cycle, and costeffectiveness. In the present review, we discuss on the possibilities of applying the CRISPR/Cas9 genome editing tool in the CVDs.

## Introduction

 Cardiovascular diseases (CVDs) remain major causes of global mortality and one of the most serious health problems in the world. The global prevalence of CVDs has risen by 93% over the last 3 decades (from 271 million in 1990 to 523 million in 2019). Moreover, the total number of deaths due to CVDs has increased about 54%, representing about one-third of all global deaths. It was demonstrated that CVD would be responsible for more than 23 million deaths (about 30.5%) by 2030 worldwide.^1,2^

 CVDs include cerebrovascular disease (stroke), heart failure, hypertensive heart disease, rheumatic heart disease, peripheral arterial disease, cardiomyopathy, and a number of other cardiac problems.^3^ Several risk factors related to the development of CVDs such as lifestyle habits and environmental factors have been identified, however, these explain only a fraction of the events. Therefore, exploration of the underlying molecular mechanisms is important for explaining cases that are not obviously related to known risk factors for the development of CVDs.^4^ It has been proven that genetic predisposition plays a pivotal role in the development of CVDs. Genetic approaches have succeeded in discovery of the molecular basis of an increasing number of cardiac diseases.^5,6^ In addition to the genes with known action in cardiovascular system, exploration of new genes associated with heart diseases may provide novel therapeutic strategies for CVDs.^6^

 Genome editing strategies are one of the most effective methods for assisting therapeutic approaches. Potential therapeutic methods of correcting disease-causing mutations or of knocking out specific genes as approaches for the prevention of CVDs have gained substantial attention using genome editing techniques.^7-9^ Videlicet, gain-of-function mutations in the pro-protein convertase subtilisin-like kexin type 9 (PCSK9) gene, which is a major regulator of low-density lipoprotein (LDL) receptor levels and LDL-cholesterol concentrations, have been reported to increase LDL-C levels, leading to an increased risk of hypercholesterolemia and coronary heart disease (CHD). In contrast, studies on loss-of-function mutations in PCSK9 indicate that inactivation of PCSK9 lowers LDL-C levels and reduces CHD, suggesting PCSK9 inhibition as a valid therapeutic method in the management of hypercholesterolemia and related diseases.^10^

 Since 1996, two kinds of designed nucleases including zinc-finger nucleases (ZFNs) and transcription activator-like effector nucleases (TALENs) have been developed. They acted as the first and second generation of gene-editing technology, respectively. Nevertheless, the high cost, low efficiency and limited accessibility have limited the application of these tools.^11^ The clustered regularly interspaced short palindromic repeats/CRISPR-associated protein 9 (CRISPR/Cas9) system, which was firstly described in 2012 by Jinek et al^12^ was developed as the third generation of gene-editing technology and it has become the most widely used genome-editing tool in molecular biology because of its benefits such as simple design, high efficiency, good repeatability, short-cycle, and cost-effectiveness.^13,14^

 In the present review, we focus on the possibilities of applying the CRISPR/Cas9 genome editing tool in the CVDs.

## Gene-Editing Mechanisms

 After finding endonuclease restriction enzymes, researchers use these enzymes for different purposes in researches. But some exciting experiments, such as genome manipulation, mutation modification, and deletion of specific genes, have always been the focus of scientists. This passion led to the development of various methods for genetic manipulation at the molecular level and gene-editing technology.

## Zinc Finger Nuclease (ZFN) Gene Editing Mechanism

 One of methods was the zinc finger nuclease method. Zinc finger transcription factors or ZF-TFs are a set of designed and engineered proteins that can attach to a specific part of DNA in a completely specific way. The protein was discovered in the study of Xenopus oocytes in 1985.^15^ ZFNs consist of two parts: The first part is zinc finger DNA-binding domains that can attach to a specific sequence of DNA. Another part is an engineered nuclease called Fok1. These two domains fuse together to form a complex that can detect a specific sequence on DNA and attach to it, using its enzymatic domain to cleavage DNA. These two domains combine to form a complex that can detect a specific sequence on DNA and attach to it, and use its enzymatic domain to cleavage DNA.

 Three factors affect the characteristics of ZFNs; the amino acid sequence that makes up each zinc finger, the number of fingers that are components of the complex, and the integration of the nuclease domain. Despite the advantages of this method, some of the factors and disadvantages of this method created limitations for the use of this method on a large scale and encouraged scientists to find alternative methods such as the high cost, time-consuming optimization of this method, and limitations in selecting target locations.^16^ Despite the advantages of this method, some of the disadvantages of this method, such as the high cost, time-consuming optimization of this method, and limitations in selecting target locations created limitations for the use of this method on a large scale and encouraged scientists to find alternative methods.^16^

## TALENs Gene Editing Mechanism

 Like zinc fingers, Transcription Activator-Like Effector Nucleases (TALENs) are made up of two different parts too, one for identifying the target site on DNA and the other for the nuclease enzyme. The domain to identify the junction on the DNA in this method is TAL effector DNA-binding, which can be designed and engineered, and another domain is an enzyme called Fok 1. The TALE part is a protein that binds to the desired location on the DNA with 33-35 amino acids. This part, depending on the type of amino acids that make it up, is able to identify and bind to a specific part of the DNA.^17^

 TALENs have better efficiency and characteristics than finger zinc. However, despite the positive features of this method, there are factors that limit the use of this method as well, including being time-consuming and the requirement of a 5‘ thymine base in target sequence.^18^

## CRISPR/Cas9 Gene-Editing Mechanism

 CRISPR/Cas9 technology consists of the two parts called Guide RNA (gRNA) and Cas9 enzyme. gRNA consists of approximately 20 nucleotides that fit into a larger RNA framework. This larger RNA framework is located on the target DNA and puts the cas9 enzyme in the right position on the DNA. The gRNA is designed as complementary to target any locus in the genome and can easily be designed by design tools including: https://wge.stemcell.sanger.ac.uk/, https://www.atum.bio/eCommerce/cas9/input,http://biotools.nubic.northwestern.edu/OligoCalc.html, http://rna.tbi.univie.ac.at/cgi-bin/RNAWebSuite/RNAfold.cgi).

**Figure 1:**
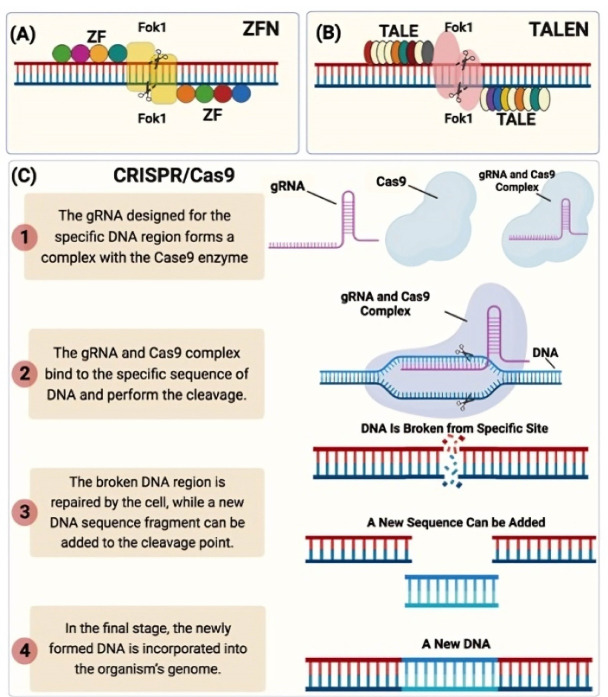
Different gene-editing technologies. (A) ZFNs contain a chain of zinc finger proteins that can bind to a specific sequence of DNA in a very specific site and by complexing with a bacterial nuclease (Fok1), can cleavage DNA strands. (B) Transcription activator-like effector (TALEs) can be designed to bind to the target DNA sequence, and this combination can cleave DNA from this specific site in association with the Fok1 nuclease enzyme. (C) Gene modification with CRISPR/Cas9 method is currently one of the strongest and best tools for gene change at the level of nucleotide sequences in DNA structure. In this figure, the mechanism of the CRISPR/Cas9 method in gene editing is shown step by step.

**Figure 2:**
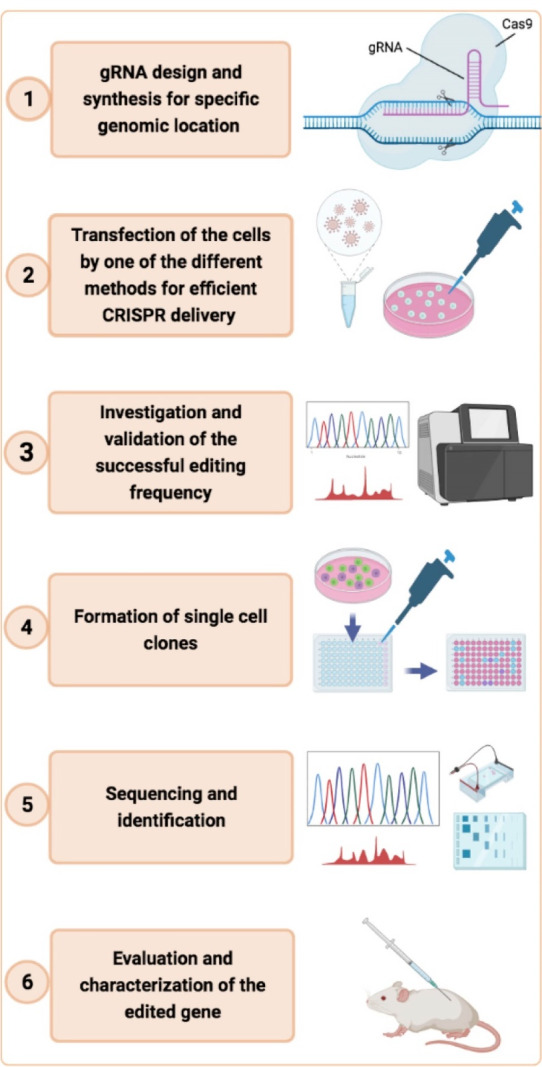
Flow chart of CRISPR/Cas9 genome editing.

 When this gRNA is added to the cas9 complex, it binds to the desired region on the DNA, making the cas9 cut point coincide with the desired point. In this way, the cut is made from the target point on the DNA. ^19^ This method is shown step by step in part (C) of Figure 1. The flow chart of CRISPR/Cas9 genome editing is presented in Figure 2.

 The same as other techniques, this method has some advantages and disadvantages. Both ZFN and TALEN have the gene-editing ability but CRISPR/Cas9 has several key advantages such as high efficiency, no drug selection required, easy delivery, and successful editing in different cell types. Both ZFN and TALEN have the gene-editing ability but CRISPR/Cas9 has several key advantages, such as high efficiency, no need for drug selection, easy delivery, and successful editing in different cell types. On the other hand, off-target cleavage is possible more frequent in CRISPR/Cas9 than TALENs and ZFNs.^20^

 Studies show that the CRISPR/Cas9 method is more efficient than other gene-editing methods. While the efficiency of CRISPR/Cas9 is 0~81%, the efficiency of the TALEN method is 0~76%, and 0~12% for ZFN. On the other hand, the possible target site is 500bp and 36bp for ZFN and TALEN, respectively, while 8 bp for CRISPR/Cas9. Also, the TALEN and ZFN methods are sensitive to Methylation, whereas the CRISPR/Cas9 method is non-sensitive. But the CRISPR/Cas9 method has more potential off-target effects than TALEN and ZFN methods. In this respect, the ZFN method has at least Off-target effects.^21^

## Basic Studies and Clinical Findings

###  In vitro models for CRISPR/Cas9

 In vitro investigations are used broadly due to this fact that they are relatively easy to maintain and manage including simplicity, species specificity, and convenience.^22^ Induced pluripotent stem cells (iPSCs) due to their close similarity to embryonic. Stem cells are the most appropriate model for assessing cardiomyogenesis on human cells. Numerus investigations have been done in the field of cardiovascular disease with patient-specific hiPSCs.^23^ These cells can be reprogrammed and also differentiated into a diversity of cells for more functional analysis. More details in the realm of cardiomyopathy, including Barth syndrome (a mitochondrial dysfunction disorder caused by mutations in the tafazzin (TAZ) gene), have been identified using Zinc Finger Nucleases (ZFNs), Transcription activator-like effector nuclease (TALEN technology) or CRISPR/Cas9 gene-editing strategy.^24,25^

 Zhang et al showed that CRISPR/Cas9 ablation of especial microRNAs disclosed their individual efficacies during differentiation of mouse embryonic stem cells. MiRNA106a, miR17 and miR93 target the cardiac suppressor gene Fog2. Fog2 is a multi-zinc finger protein, which is associates with a cardiac transcription factor, GATA-4. GATA-4 is required for normal heart development as well hypertrophic responses in cardiac myocytes.^26^ In a human in vitro cardiac model, researchers suggested that KCNQ1-SupRep gene therapy by CRISPR-Cas9 in induced pluripotent stem cell-derived cardiomyocytes (iPSC-CMs) can be considered for complete correction of long QT syndrome.^27^ Similarly, Yamamoto et al^28^ using Cas9 double nickase system in hiPSCs, generate in vitro allele-specific knockout models of channelopathy long QT syndrome (LQTS).

 Generally, based on in vitro evidences, CRISPR/Cas9 shows great potency for future applications in in vivo and human studies.

###  In vivo models for CRISPR/Cas9

 CRISPR/Cas9 has been applied to a different small animal including zebrafish, rats, mice or large animal such as pigs. Some mutations which are responsible for cardiomyopathies including dilated cardiomyopathy, Barth syndrome, long-QT syndrome, hypertrophic cardiomyopathy (HCM), and Duchenne muscular dystrophy (DMD) have been corrected by genome editing in patient-specific iPSC derived cardiomyocytes.^29-31^

 Drug resistance remains as a challenge in the treatment of Proprotein convertase subtilisin/kexin type 9 (PCSK9) 2-overexpressed low-density lipoprotein (LDL). Using CRISPR/Cas9 genome editing, Ding et al^32^ reported a loss of function for the PCSK9 gene in the livers of mice and consequently a decrease of the cholesterol levels by over 40%. In another study, inhibiting several genes function including Apolipoprotein E (ApoE), cluster of differentiation 36 (CD36), LDL receptor, leptin, and ryanodine receptor type 2 (RyR2) using RNA-guided Cas9 nucleases were represented.^33^ A mice model of DMD enhanced skeletal muscle function 4 weeks after IM-adeno-associated virus–9 (AAV9)-Cas9 injection.^34^

 Also, Mendell et al^35^ have shown that CRISPR/Cas9 -treated mice significantly improved muscle function via clavation, repairing or removing faulty exon of the dystrophin gene. Dysfunctional of ryanodine receptor type 2 (RYR2) are responsible for approximately 60% of all catecholaminergic polymorphic lethal ventricular tachycardia. In an animal model researchers indicated that adeno-associated virus (AAV) serotype 9-based delivery of the Cas9 system can efficiently edit cardiomyocytes through specifically targeting the disease-causing allele.^36^ Interestingly, other *in vivo* model indicated that AAV-CRISPR/Cas9–mediated Ldlr gene correction can ameliorate atherosclerosis phenotypes and be a potent treatment strategy for patients with familial hypercholesterolemia.^37^

 Overall, based on the results of the *in vivo* studies, CRISPR/Cas9-mediated gene editing system is a promising strategy to alter the function of genes connected to CVDs.

###  Prospective application for CRISPR/Cas9 in clinical human studies

 CRISPR genome-editing technology seems to be worth watching for both researchers and clinicians. Human CRISPR/Cas9 clinical trials received ethical approval in China and the United States^38^. Recent evidences claimed this strategy as a novel treatment for several genetic disorders, including some of cancers, neurodegenerative diseases, sickle cell anemia, Duchenne muscular dystrophy, viral infections, immune disorders, cystic fibrosis, and cardiovascular diseases.^39-42^ Various studies have supported this hypothesis that, the combination of genome-wide association studies (GWAS) and CRISPR genome engineering strategy could play an important role in the development of human personalized medicine.^42^

**Figure 3:**
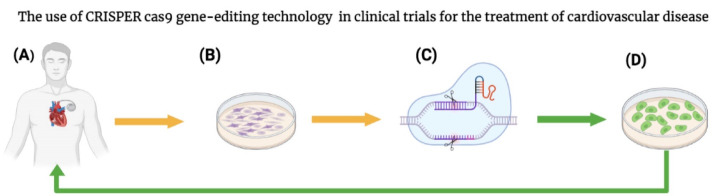
Use of CRISPR Cas9 gene- editing technology in clinical trials for the treatment of cardiovascular disease (A) sample is taken from a patient with cardiovascular disease. (B) Cardiovascular cells are isolated from the patient. (C) Corrections are made in cells with the CRISPR cas9 method. (D) The patient-specific corrected cells are transplanted into the patient.

 In a recent trial, researchers investigated the use of CRISPR/Cas9–based gene editing for treating two patients with inherited diseases: one patient with transfusion-dependent β-thalassemia (TDT) and the other in a patient with and sickle cell disease (SCD). Based on their results, both patients had early, substantial, and sustained increases in fetal hemoglobin levels after the administration of CTX001, with more than 99% pancellularity during a 12-month period. Along with the reported advantages, some adverse events were documented in both patients such as pneumonia in the presence of neutropenia, sepsis in the presence of neutropenia, cholelithiasis, veno-occlusive liver disease with sinusoidal obstruction syndrome (VOD–SOS), and abdominal pain after TX001 infusion. Next step, the administration of CTX001 to additional eight patients (six with TDT and two with SCD) was done. Their results supported further experimental testing of CRISPR/Cas9 gene-editing approaches for treating genetic diseases.^43^ The use of CRISPR/Cas9 gene-editing technology in clinical trials for the treatment of cardiovascular disease Shown schematically in Figure 3.

## CRISPR/Cas9: future perspectives, concerns and its application in heart disease

 It is a fact that this method has promising potential for treating diseases. Cardiovascular diseases caused by a genomic defect constitute potential candidates for treatment with this method. However, research with this method is limited to in vitro and animal models only. If these researches continue, treatment methods at the gene level for different heart diseases will likely emerge. However, these experiments were limited due to some ethical problems. Can editing human fetal cells at the genome level be ethical? This issue is open to discussion. However, it is a fact that some may use this technology for non-therapeutic purposes, and this necessitates the ethical use of this technology.^44^

## Conclusion

 Collectively, recent structural and mechanistic studies on the realm of CRISPR/Cas9 genome editing technology in in vitro, in vivo and human studies open new therapeutic perspectives for treating cardiovascular diseases. However, for broad using of this method for human studies, some points need to be considered. Firstly, since the SpCas9 and SaCas9 proteins are the most commonly used Cas9 proteins, the major delivery challenge in terms of packaging into AAV due to their large size must be resolved based on this fact. Discovering smaller Cas9 orthologs or reducing the size of the SpCas9 and SaCas9 proteins can be a strong point for solving this limitation. Furthermore, more characterization and optimization are needed in order to its therapeutic application. ^45^ Beyond that, despite the suggestive application of CRISPR technology including; genome editing, endogenous genes expression, epigenome editing, and edition of RNA, several challenges should be targeted in future studies.

 In Total, the unconquerable limitation of the CRISPR/Cas9 editing system is the variability in its efficiency and potential off-target gene editing. In addition, germline editing by this technique, mainly in humans, raises societal and ethical considerations.^46^

## Acknowledgments

 The authors are thankful to the Cardiovascular Research Center, Tabriz University of Medical Sciences.

## Funding

 None.

## Competing interests

 Authors state no conflict of interest.

## References

[1] Roth GA, Mensah GA, Johnson CO, Addolorato G, Ammirati E, Baddour LM (2020). Global burden of cardiovascular diseases and risk factors, 1990-2019: update from the GBD 2019 study. J Am Coll Cardiol.

[2] Amini M, Zayeri F, Salehi M (2021). Trend analysis of cardiovascular disease mortality, incidence, and mortality-to-incidence ratio: results from Global Burden of Disease Study 2017. BMC Public Health.

[3] Murray CJL, Aravkin AY, Zheng P, Abbafati C, Abbas KM, Abbasi-Kangevari M (2020). Global burden of 87 risk factors in 204 countries and territories, 1990-2019: a systematic analysis for the Global Burden of Disease Study 2019. Lancet.

[4] Lara-Pezzi E, Dopazo A, Manzanares M (2012). Understanding cardiovascular disease: a journey through the genome (and what we found there). Dis Model Mech.

[5] Kathiresan S, Srivastava D (2012). Genetics of human cardiovascular disease. Cell.

[6] Khera AV, Kathiresan S (2017). Genetics of coronary artery disease: discovery, biology and clinical translation. Nat Rev Genet.

[7] Li H, Yang Y, Hong W, Huang M, Wu M, Zhao X (2020). Applications of genome editing technology in the targeted therapy of human diseases: mechanisms, advances and prospects. Signal Transduct Target Ther.

[8] Priori SG, Barhanin J, Hauer RN, Haverkamp W, Jongsma HJ, Kleber AG (1999). Genetic and molecular basis of cardiac arrhythmias: impact on clinical management parts I and II. Circulation.

[9] Alipour S, Sakhinia E, Khabbazi A, Samadi N, Babaloo Z, Azad M (2020). Methylation status of interleukin-6 gene promoter in patients with Behçet’s disease. Reumatol Clin (Engl Ed).

[10] Bergeron N, Phan BA, Ding Y, Fong A, Krauss RM (2015). Proprotein convertase subtilisin/kexin type 9 inhibition: a new therapeutic mechanism for reducing cardiovascular disease risk. Circulation.

[11] Kim YG, Cha J, Chandrasegaran S (1996). Hybrid restriction enzymes: zinc finger fusions to Fok I cleavage domain. Proc Natl Acad Sci U S A.

[12] Jinek M, Chylinski K, Fonfara I, Hauer M, Doudna JA, Charpentier E (2012). A programmable dual-RNA-guided DNA endonuclease in adaptive bacterial immunity. Science.

[13] Xu Y, Li Z (2020). CRISPR-Cas systems: overview, innovations and applications in human disease research and gene therapy. Comput Struct Biotechnol J.

[14] Zaib S, Saleem MA, Khan I (2022). CRISPR-Cas9 genome engineering: trends in medicine and health. Mini Rev Med Chem.

[15] Diakun GP, Fairall L, Klug A (1986). EXAFS study of the zinc-binding sites in the protein transcription factor IIIA. Nature.

[16] Li H, Yang Y, Hong W, Huang M, Wu M, Zhao X (2020). Applications of genome editing technology in the targeted therapy of human diseases: mechanisms, advances and prospects. Signal Transduct Target Ther.

[17] Bogdanove AJ, Voytas DF (2011). TAL effectors: customizable proteins for DNA targeting. Science.

[18] Cebrailoglu N, Yildiz AB, Akkaya O, Ciftci YO (2019). CRISPR-Cas: removing boundaries of the nature. Eur J Biol.

[19] Cao G, Xuan X, Zhang R, Hu J, Dong H (2021). Gene therapy for cardiovascular disease: basic research and clinical prospects. Front Cardiovasc Med.

[20] Walsh RM, Hochedlinger K (2013). A variant CRISPR-Cas9 system adds versatility to genome engineering. Proc Natl Acad Sci U S A.

[21] Chen L, Tang L, Xiang H, Jin L, Li Q, Dong Y (2014). Advances in genome editing technology and its promising application in evolutionary and ecological studies. Gigascience.

[22] Smith AJP, Deloukas P, Munroe PB (2018). Emerging applications of genome-editing technology to examine functionality of GWAS-associated variants for complex traits. Physiol Genomics.

[23] Musunuru K, Sheikh F, Gupta RM, Houser SR, Maher KO, Milan DJ (2018). Induced pluripotent stem cells for cardiovascular disease modeling and precision medicine: a scientific statement from the American Heart Association. Circ Genom Precis Med.

[24] Motta BM, Pramstaller PP, Hicks AA, Rossini A (2017). The impact of CRISPR/Cas9 technology on cardiac research: from disease modelling to therapeutic approaches. Stem Cells Int.

[25] Moscou MJ, Bogdanove AJ (2009). A simple cipher governs DNA recognition by TAL effectors. Science.

[26] Zhang Z, Ursin R, Mahapatra S, Gallicano GI (2018). CRISPR/CAS9 ablation of individual miRNAs from a miRNA family reveals their individual efficacies for regulating cardiac differentiation. Mech Dev.

[27] Dotzler SM, Kim CSJ, Gendron WAC, Zhou W, Ye D, Bos JM (2021). Suppression-replacement KCNQ1 gene therapy for type 1 long QT syndrome. Circulation.

[28] Yamamoto Y, Makiyama T, Harita T, Sasaki K, Wuriyanghai Y, Hayano M (2017). Allele-specific ablation rescues electrophysiological abnormalities in a human iPS cell model of long-QT syndrome with a CALM2 mutation. Hum Mol Genet.

[29] Hinson JT, Chopra A, Nafissi N, Polacheck WJ, Benson CC, Swist S (2015). HEART DISEASE. Titin mutations in iPS cells define sarcomere insufficiency as a cause of dilated cardiomyopathy. Science.

[30] Chavali NV, Kryshtal DO, Parikh SS, Wang L, Glazer AM, Blackwell DJ (2019). Patient-independent human induced pluripotent stem cell model: a new tool for rapid determination of genetic variant pathogenicity in long QT syndrome. Heart Rhythm.

[31] Karakikes I, Ameen M, Termglinchan V, Wu JC (2015). Human induced pluripotent stem cell-derived cardiomyocytes: insights into molecular, cellular, and functional phenotypes. Circ Res.

[32] Ding Q, Strong A, Patel KM, Ng SL, Gosis BS, Regan SN (2014). Permanent alteration of PCSK9 with in vivo CRISPR-Cas9 genome editing. Circ Res.

[33] Yang D, Xu J, Zhu T, Fan J, Lai L, Zhang J (2014). Effective gene targeting in rabbits using RNA-guided Cas9 nucleases. J Mol Cell Biol.

[34] Long C, Amoasii L, Mireault AA, McAnally JR, Li H, Sanchez-Ortiz E (2016). Postnatal genome editing partially restores dystrophin expression in a mouse model of muscular dystrophy. Science.

[35] Mendell JR, Rodino-Klapac LR (2016). Duchenne muscular dystrophy: CRISPR/Cas9 treatment. Cell Res.

[36] Pan X, Philippen L, Lahiri SK, Lee C, Park SH, Word TA (2018). In vivo RyR2 editing corrects catecholaminergic polymorphic ventricular tachycardia. Circ Res.

[37] Zhao H, Li Y, He L, Pu W, Yu W, Li Y (2020). In vivo AAV-CRISPR/Cas9-mediated gene editing ameliorates atherosclerosis in familial hypercholesterolemia. Circulation.

[38] Long C, Li H, Tiburcy M, Rodriguez-Caycedo C, Kyrychenko V, Zhou H (2018). Correction of diverse muscular dystrophy mutations in human engineered heart muscle by single-site genome editing. Sci Adv.

[39] Barrangou R, Doudna JA (2016). Applications of CRISPR technologies in research and beyond. Nat Biotechnol.

[40] Dominguez AA, Lim WA, Qi LS (2016). Beyond editing: repurposing CRISPR-Cas9 for precision genome regulation and interrogation. Nat Rev Mol Cell Biol.

[41] Mani I (2021). Genome editing in cardiovascular diseases. Prog Mol Biol Transl Sci.

[42] Torres-Ruiz R, Rodriguez-Perales S (2017). CRISPR-Cas9 technology: applications and human disease modelling. Brief Funct Genomics.

[43] Meisel R (2021). CRISPR-Cas9 gene editing for sickle cell disease and β-thalassemia. N Engl J Med.

[44] Neldeborg S, Lin L, Stougaard M, Luo Y (2019). Rapid and efficient gene deletion by CRISPR/Cas9. Methods Mol Biol.

[45] Schreurs J, Sacchetto C, Colpaert RMW, Vitiello L, Rampazzo A, Calore M (2021). Recent advances in CRISPR/Cas9-based genome editing tools for cardiac diseases. Int J Mol Sci.

[46] Daley GQ, Lovell-Badge R, Steffann J (2019). After the storm-a responsible path for genome editing. N Engl J Med.

